# Transcriptomic profiling of single circulating tumor cells provides insight into human metastatic gastric cancer

**DOI:** 10.1038/s42003-021-02937-x

**Published:** 2022-01-11

**Authors:** Ryo Negishi, Hitomi Yamakawa, Takeru Kobayashi, Mayuko Horikawa, Tatsu Shimoyama, Fumiaki Koizumi, Takeshi Sawada, Keisuke Oboki, Yasushi Omuro, Chikako Funasaka, Akihiko Kageyama, Yusuke Kanemasa, Tsuyoshi Tanaka, Tadashi Matsunaga, Tomoko Yoshino

**Affiliations:** 1 Division of Biotechnology and Life science, Institute of Engineering, Tokyo University of Agriculture and Technology, 2-24-16, Naka-cho, Koganei, Tokyo, 184-8588 Japan; 2 Department of Medical Oncology, Tokyo Metropolitan Cancer and Infectious Diseases Center Komagome Hospital, Tokyo, Japan; 3 Department of Laboratory Medicine, Tokyo Metropolitan Cancer and Infectious Diseases Center Komagome Hospital, Tokyo, Japan; 4 Center for Medical Research Cooperation, Tokyo Metropolitan Institute of Medical Science, Setagaya‐ku, Japan

**Keywords:** Cancer genetics, Metastasis

## Abstract

Transcriptome analysis of circulating tumor cells (CTCs), which migrate into blood vessels from primary tumor tissues, at the single-cell level offers critical insights into the biology of metastasis and contributes to drug discovery. However, transcriptome analysis of single CTCs has only been reported for a limited number of cancer types, such as multiple myeloma, breast, hepatocellular, and prostate cancer. Herein, we report the transcriptome analysis of gastric cancer single-CTCs. We utilized an antigen-independent strategy for CTC isolation from metastatic gastric cancer patients involving a size-dependent recovery of CTCs and a single cell isolation technique. The transcriptomic profile of single-CTCs revealed that a majority of gastric CTCs had undergone epithelial-mesenchymal transition (EMT), and indicated the contribution of platelet adhesion toward EMT progression and acquisition of chemoresistance. Taken together, this study serves to employ CTC characterization to elucidate the mechanisms of chemoresistance and metastasis in gastric cancer.

## Abstract (web summary)

Ryo Negishi et al. utilize a size-dependent recovery method with gel-based cell manipulation to isolate single circulating tumor cells (CTCs) for transcriptomic analysis. They demonstrate the utility of this approach by analyzing the transcriptomic profile of CTCs from human gastric cancer patients.

## Introduction

Circulating tumor cells (CTCs) are cells that migrate into blood vessels from the primary tumor site and are involved in the metastasis of cancer to distant tissues. CTCs are the main targets of liquid biopsy along with circulating tumor DNA (ctDNA) and exosomes^1,2^. Unlike ctDNA and exosomes, CTCs have an advantage in that they directly provide information on the metabolism and signaling of cancer cells. CTCs are used for genome-wide mutation^3^ and transcriptome analysis^4^, as well as cell culture^5,6^, and could be a non-invasive source of a vast amount of cellular information on tumor composition, invasiveness, drug susceptibility, and resistance to therapy. CTCs are expected to be used not only for cancer diagnosis but also for the development of therapeutic drugs^7^.

Recently, transcriptome analysis of CTCs identified candidate genes associated with metastasis and signal pathways associated with resistance to chemotherapy^8,9^. Transcriptome analysis of CTCs at the single-cell level has shown that CTCs are a heterogeneous population between and within patient groups. This may be due to cells undergoing epithelial-mesenchymal transition (EMT); both epithelial and mesenchymal cell types have been identified in CTCs^10,11^. It has also been shown that EMT-transformed CTCs have concurrent cancer stem cell-like properties^12^. Transcriptome analysis of CTCs at a single-cell level is considered to be a powerful tool for obtaining critical insights, such as response to treatment, that cannot be acquired by analyzing cancer tissues or by in vitro experiments^7^. In contrast, transcriptome analysis of single CTCs has only been reported in small-scale clinical trials for a limited number of cancer types, such as breast^10,13–15^, hepatocellular^16,17^, and prostate cancer^7,18,19^ and multiple myeloma^20,21^, and there is a need to expand this investigation to a variety of cancer types.

Gastric cancer is the third leading cause of death from cancer worldwide^22^, with a majority of patients concentrated in the Asia-Pacific region and developing countries. In particular, metastatic gastric cancer is known as a very diverse cancer type with a 5-year survival rate of <10%^23^. Gastric cancer has a high recurrence rate after resection^24^, and most cases of recurrence and metastasis are refractory to chemotherapy^25^. The CellSearch system, using EpCAM antibodies, is the gold standard for measuring CTCs in other cancer types; it has been reported to be effective in predicting prognosis and risk of recurrence for gastric cancer^26,27^. Notably, the number of EpCAM-positive CTCs is lower in gastric cancer with a lower cut-off value compared to other cancer types^28^. It has been noted that as a result of EMT, it is difficult to detect gastric cancer CTCs with mesenchymal phenotypes^28,29^. Recently, RNA-ISH analysis of gastric CTCs showed the presence of mesenchymal CTCs and revealed their potential for monitoring therapy response^30^. However, the study defined mesenchymal CTCs using only two genes (*vimentin and twist*) and underlying mechanism of EMT in gastric CTCs were still unknown. Till date, there have been no reports on the transcriptome analysis of gastric cancer CTCs as described above. Therefore, a single-CTC transcriptome analysis, including EMT-induced CTCs, is essential to elucidate the properties of gastric CTCs.

In this study, we aimed to characterize gastric cancer CTCs by single-cell isolation and transcriptome analysis of CTCs from cases of metastatic gastric cancer. Our research group has established the microcavity array (MCA)/gel-based cell manipulation (GCM) method, a size-dependent recovery of CTCs using microfilters and single cell isolation technique, which can be applied to single-cell genome analysis^31,32^. Recent commercialized single-cell isolation system (Droplet-based system, nanowell-based system and fluorescence activated cell sorting) is focused to isolate single-cells from cell population by random; therefore, it is difficult to isolate rare single cell such as CTCs. Some systems such as VyCap Puncher^33^, DEPArray^34^, ALS CellColector^35^, and RareCytes CyteFinder^36^ system have been developed for isolation of rare single CTCs. However, these systems are not integrated with the CTC enrichment system, necessitating CTC enrichment through other systems. This step may pose a risk of cell loss during sample handling. Although the RareCytes CytoFinder system was designed to perform the complete process of CTC isolation (CTC enrichment, staining, detection, and single-cell isolation), there is a risk of contamination of white blood cells because enriched cells were positioned at random. The MCA/GCM system was designed to perform the complete process of CTC isolation. Moreover, MCA can entrap single cells at even intervals; this allows isolation of single cells at high efficiency and without any contamination^32^. In this study, we separated gastric cancer CTCs by the surface antigen-independent MCA/GCM method and constructed a pipeline that enables RNA-seq analysis of single CTCs. Furthermore, our proposed method revealed the heterogeneity of the transcriptome of gastric cancer CTCs and characterized the gene expression pattern of EMT-induced CTCs that could reveal the underlying mechanism of EMT regulation in gastric cancer. The results of this study will also help to elucidate the mechanisms involved in the acquisition of drug resistance and metastasis of CTCs.

## Results

### Performance evaluation of the GCM-based method in single-cell whole transcriptome amplification (WTA)

**Fig. 1:**
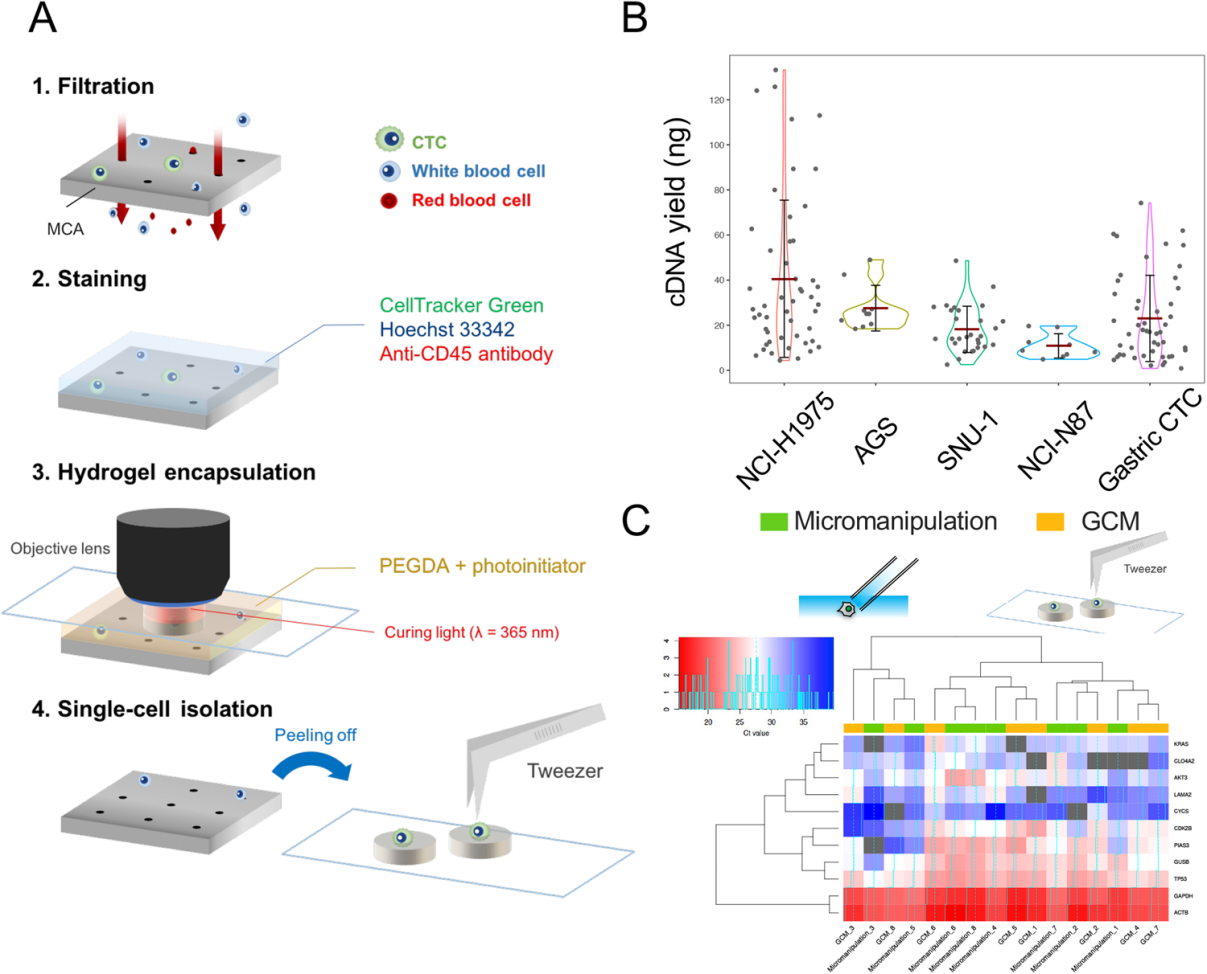
Single-cell isolation and gene expression analysis based on microcavity array and gel-based cell manipulation. **A** Schematic overview of single-cell isolation based on MCA/GCM; **B** Violin plot shows the yield of WTA products from cancer cell lines and gastric CTCs. Each dot represents a single-cell. The red bar represents mean value. The black bar represents deviation. **C** Cluster analysis of single-cell gene expression patterns. Heat map shows Cq values of 11 genes by qPCR assays performed on single NCI-H1975 cells isolated by micromanipulation (green) and MCA/GCM (yellow). Red indicates high gene expression, white indicates median gene expression, blue indicates low gene expression, and gray represents undetectable expression.

Figure 1A showed schematic images of the GCM-based single-cell isolation method^32^. CTCs on the MCA were processed by RNA stabilization solution (CellCover) and stained with CellTracker Green and fluorescence-labelled antibody for CD45. Then, photopolymerizable hydrogel prepolymer was mounted and single cells were encapsulated into pillar-shaped hydrogels by light irradiation under the fluorescence microscope. Hydrogels were recovered by tweezers and subjected to subsequent single-cell WTA.

Prior to clinical trials, WTA of hydrogel-encapsulated single cells was performed using gastric cancer cell lines (SNU-1, NCI-N87, and AGS) and lung cancer cell line (NCI-H1975) to evaluate the performance of the MCA/GCM-based method. Although the cDNA yield varied depending on the cell lines, sufficient yield of cDNA from all cell lines were obtained for subsequent RNA-seq analysis (>10 ng/assay) (Fig. 1B). The average length of cDNA ranged from 823 to 1360 bp (Supplementary Fig. 1), indicating that sufficient cDNA length for gene expression analysis was obtained^37^. The amount of cDNA in lung cancer cell lines was relatively higher than that of gastric cancer cell lines (Fig. 1B). The WTA yields were proportional to the initial amount of mRNA in Quartz-Seq (Supplementary Fig. 2). A recent report suggests that mRNA amount in a single cell depends on the cell size^38^. The size of lung cancer cell line (NCI-H1975: 23.5 μm) was relatively larger than gastric cancer cells (AGS: 14.9 μm, SNU-1: 11.7 μm, NCI-N87: 12.2 μm), therefore, the initial amount of mRNA may be higher in lung cancer cells compared to gastric cancer cells, resulting in a higher yield of the resulting cDNA in WTA.

Next, we compared the GCM-based method and micromanipulation in terms of the yield and the average length of cDNA in WTA of lung cancer cell line NCI-H1975 cells. The cDNA yield in GCM was higher than that obtained by micromanipulation (Supplementary Fig. 3A), whereas no significant difference was observed in the average length (Supplementary Fig. 3B, *p* = 0.147). Because the sample volume in single-cell Quartz-seq (WTA) is extremely low (0.4 μl), the reaction is largely unaffected by excess water. Excess water cannot be avoided during micromanipulation as the single-cell sample volume is transferred to a reaction tube, and lower cDNA yield in micromanipulation may be due to the water content in the WTA reaction. In other words, GCM-based single-cell manipulation is advantageous for extremely low volume reactions because hydrogel-encapsulated single-cells comprise of minimal water.

**Fig. 2:**
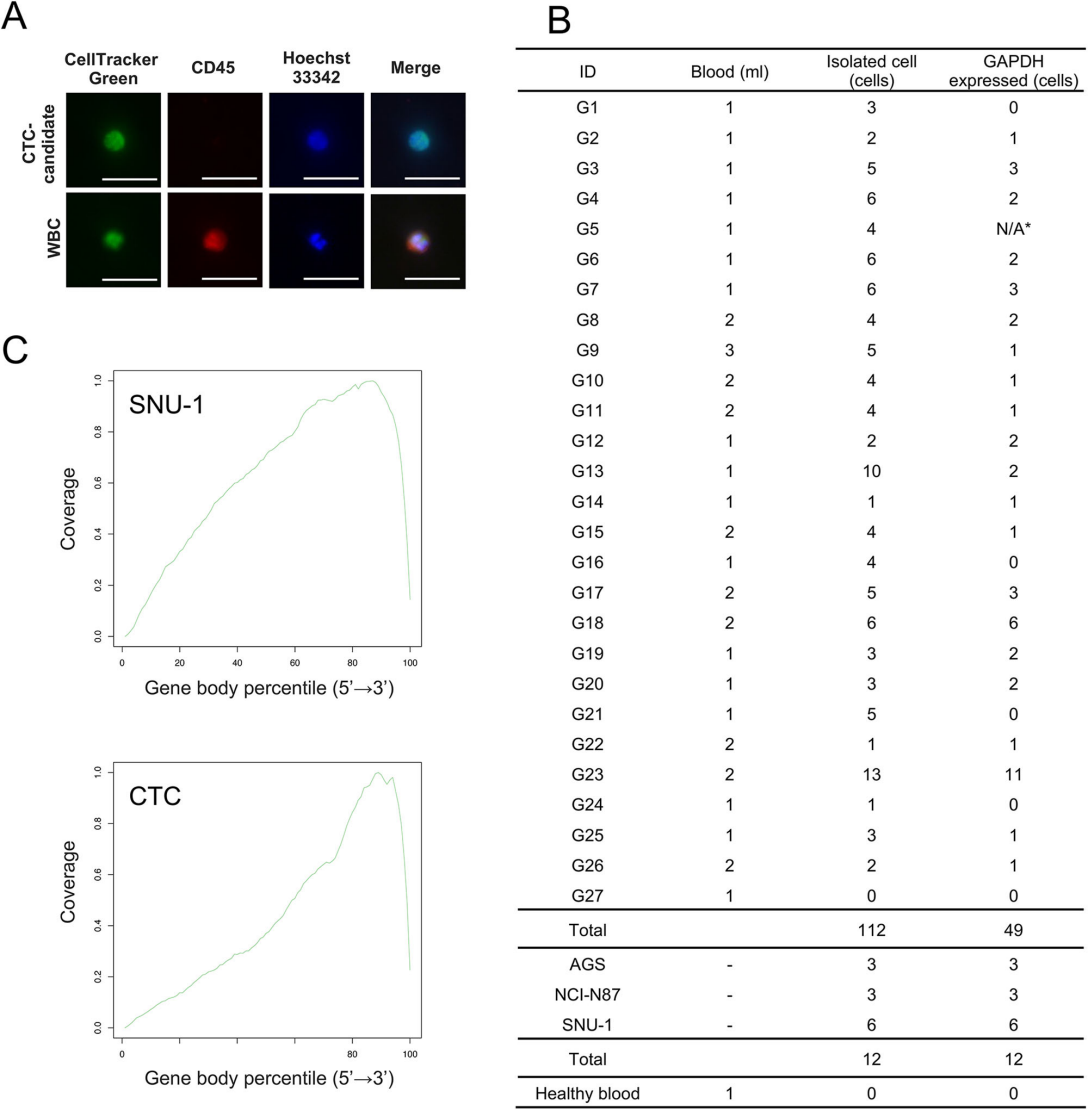
Isolation of gastric CTCs and RNA-seq analysis. **A** Fluorescence image of a representative CTC-candidate and WBC. Scale bar: 50 μm. **B** Summary of CTC isolation and quality control. Samples in G5 were lost by technical error (N/A*). **C** Gene body coverage from a single SNU-1 cell and gastric CTC.

Furthermore, gene expression patterns of single-cells in the GCM-based method and micromanipulation were compared to assess WTA bias. Quantitative PCR (qPCR) analysis for 11 genes (including two housekeeping genes: *GAPDH* and *ACTB*) was carried out on the whole transcriptomes of NCI-H1975 cells (eight single-cells each). Figure 1C shows the results of cluster analysis on gene expression patterns of a total of 16 single-cells. Gene expression was represented as Cq values: red, high; white, median; blue, low; gray, undetectable gene expression. Cluster analysis revealed no difference in gene expression patterns between the GCM-based method and micromanipulation as no clustering appeared by either method. These results indicate that the GCM-based method exhibits a similar performance as micromanipulation in gene expression analysis. Besides, we evaluated the gene expression of *GAPDH* of GCM-isolated single gastric cancer cell lines (*n* = 12) for the quality control (QC) for single-cell RNA-seq analysis. The *GAPDH* expression was successfully detected from all of gastric cancer cell lines (Fig. 2B).

### Isolation and characterization of CTC candidates from metastatic cancer patients

Blood samples were collected from 27 gastric cancer patients between April 2017 and October 2019 (Supplementary Data 1). Out of these samples, 112 CTC candidates from 26 patients were detected by immunostaining (Fig. 2A & B) and were subjected to WTA. Besides, we also performed CTC isolation test for one healthy donor and did not detect any CTC candidates (Fig. 2B). The yield of amplified cDNA from single-CTC candidates ranged from 0.1 to 99.3 ng (Fig. 1B). A large variation of CTC cDNA yield was observed compared to gastric cancer cell lines. Previously described, cDNA yield was affected by input amount of RNA. Besides, CTCs didn’t originate from single cancer cell, unlike cancer cell line. Thus, these results indicated CTCs might be cell population with a wide range of mRNA abundance compared to cell lines. Then, the gene expression of *GAPDH* was evaluated as a QC for cDNA prior to RNA-seq (Fig. 2B). The 49 cells that passed the QC were defined as CTC candidates with sufficient quality RNA. The QC pass rate of CTCs was 43.8% (49/112 CTCs).

### Evaluation of quality and contents of RNA-seq data

**Fig. 3:**
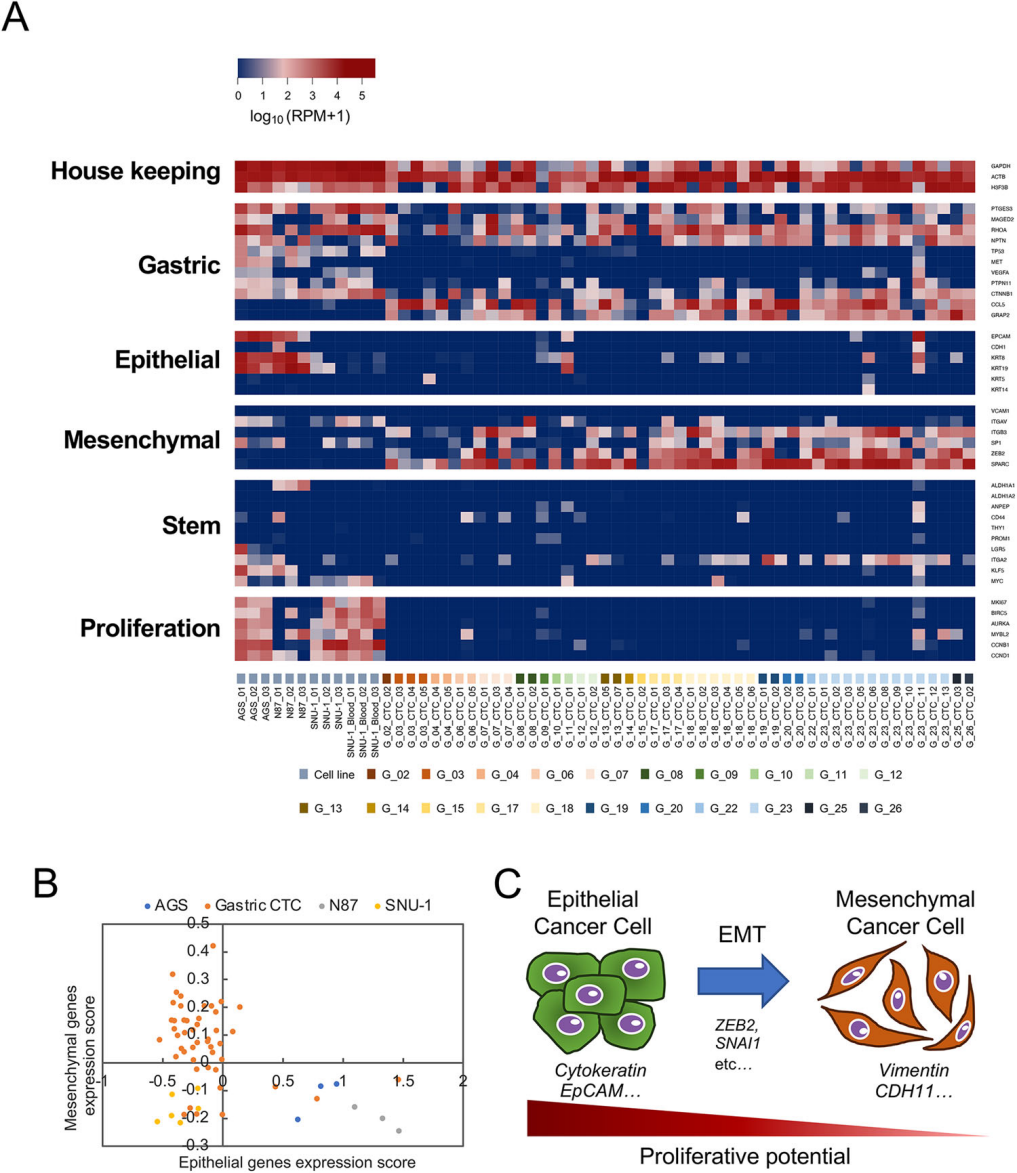
Targeted analysis of single-cell RNA-seq data. **A** The heat map shows expression of housekeeping genes, gastric, epithelial, epithelial, stem cell, and proliferation markers. Scale in log10 (RPM + 1). Expression level increases from blue to red. **B** Scatter plots based on the expression levels of epithelial and mesenchymal genes. **C** Schematic of EMT induction and proliferative potential of cancer cells.

We performed RNA-seq analysis for 49 single CTC-candidates, three AGS cells (gastric cancer cell line), three NCI-N87 cells (gastric cancer cell line), and six SNU-1 cells (gastric cancer cell line, three cells were isolated from PBS, others were isolated from cancer cell-spiked bloods). Approximately 5 M raw reads were obtained from 49 CTC-candidates and 12 single cancer cells. The alignment rate of raw reads from single CTC-candidates to the human genome were 68.9 ± 17.4%, and was comparable to the raw reads from single cancer cells (73.8 ± 22.9%). Figure 3C shows the distribution of mapped reads of single-cell RNA-seq data. Although SNU-1 showed 3′ bias due to poly(A) tailing-based WTA, data from CTC-candidates showed a stronger 3′ bias. These data suggested that RNA in CTCs tended to be degraded, therefore, we chose RPM as a gene expression unit. To evaluate transcript abundance, we designated transcripts with RPM <1.0 as detectable transcripts. We detected 4376–12,084 transcripts from single cancer cells (*n* = 12), whereas, 4064 ± 1967 transcripts were detected from single CTC-candidates (with a range of 1183 to 11,230 transcripts, *n* = 49). Besides, there was no correlation between the number of detected transcripts and the yield of amplified cDNA. We evaluated the gene expression value of the WBC marker CD45 (*PTPRC*) to detect contaminated WBCs. Almost all CTC candidates (*n* = 48) were negative for the WBC marker CD45 (*PTPRC*). One CTC candidate (G_18_CTC_4) showed weak expression of the *PTPRC* gene. However, we did not exclude these cells from gene expression analysis because their expression levels were very low. Therefore, we confirmed that CTC candidates were not contaminated with WBCs. In addition, we measured the ratio of mitochondrial gene expression to evaluate the quality of the single-cell library (Supplementary Fig. 4). The ratio of mitochondrial genes of CTC-candidates was range from 0.02 to 68.17% (average: 5.07%; median: 1.33%). We excluded two single cells (G_04_CTC_05 and G_07_CTC_03) with >25% mitochondrial gene ratio as low-quality cells from further analysis.

### Expression patterns of gene markers in single-CTCs

To characterize gastric cancer CTCs, we first evaluated the expression patterns of known markers. Figure 3A illustrates the expression of housekeeping genes, gastric cancer gene markers, epithelial marker genes, mesenchymal marker genes, and stem cell/cell proliferation-related genes^10,12,39–41^. Blue color indicates low expression level of genes, whereas red represents high expression level. *RHOA* or *CTNNB1* was expressed in all CTCs (47/47). Analysis of CTCs in prostate cancer and melanoma also shows that CTCs retain the gene expression pattern characteristic of the primary tumor^18,42^. Thus, gastric cancer CTCs have been suggested to possess characteristics of gastric cancer cells. As expected, almost all CTCs displayed low expression of epithelial marker genes (*KRT* family and *EpCAM*) and high expression of mesenchymal genes such as *ZEB2*, which is one of the widely known EMT-inducing factors, and *SERPINE1*. Furthermore, 23 CTCs expressed epithelial genes such as the KRT family of genes (Fig. 3A). To evaluate the EMT signature of CTCs, we measured the gene expression scores of epithelial and mesenchymal genes (Fig. 3B). Most CTCs showed stronger expression of mesenchymal genes than the cell lines. However, three CTCs showed expression of epithelial genes that was comparable with the expression observed in epithelial gastric cancer cell lines (AGS and N87). Furthermore, two CTCs showed weak expression of epithelial genes, despite expressing mesenchymal genes. These results indicate that most gastric cancer CTCs had undergone EMT, consistent with the results of previous reports^43,44^. However, the expression of proliferation markers in CTCs was much lower than the cell lines. In particular, the expression of *Ki67* (*MKI67*), which has an integral role in cell cycle progression, was not detected, suggesting that CTCs are a cell population in which growth has been arrested. In general, mesenchymal cancer cells have lower proliferative potential than epithelial cancer cells (Fig. 3C)^45^. This low proliferative potential of CTCs may be due to EMT. Biphenotypic properties and arrested cell proliferation have been confirmed in CTCs of pancreatic, prostate, and breast cancer^7,12,13^. These characteristics may also provide CTCs with important phenotypes for survival in blood vessels. In addition, expression of stem cell markers such as *CD44* was observed in 16% (8/47) CTCs and *ITGA2* in 45% (22/47) CTCs. This result suggested that a portion of gastric cancer CTCs have a cancer stem cell-like property. Besides, *ITGA2* is not only a mesenchymal stem cell marker, but also a protein that contributes to binding to extracellular matrix and platelets. Recent studies on cancer tissues suggest that *ITGA2* is related to the invasiveness of gastric and pancreatic cancer cells^46,47^. This result suggests that some external factors may influence CTCs and that gastric cancer CTCs have heterogeneous properties.

### Cluster analysis of single-CTCs and clinical relevance

**Fig. 4:**
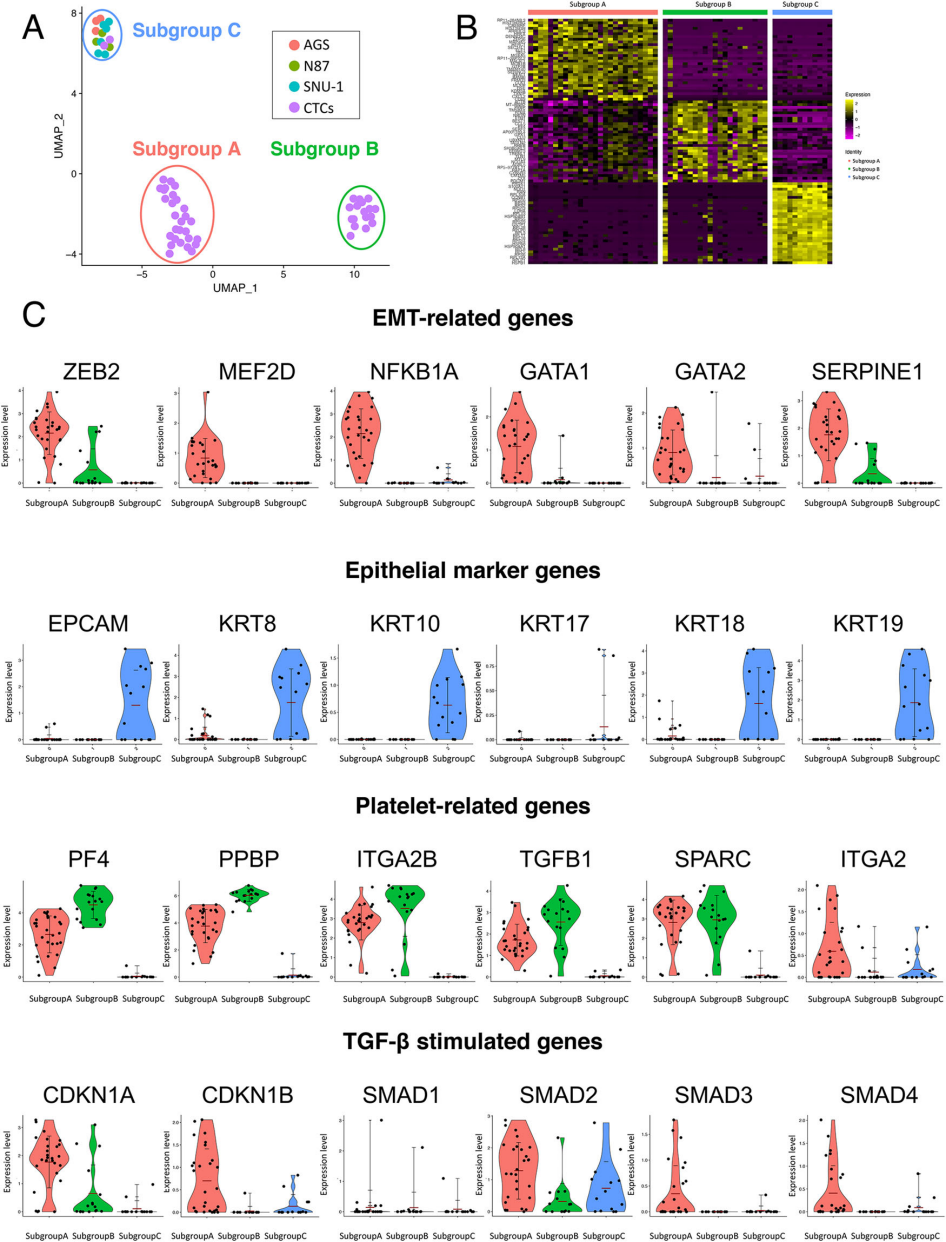
Gene expression and clustering of gastric CTCs. **A** UMAP plot of single gastric cancer cells and gastric CTCs. (12 cancer cells and 47 CTCs from 21 patients. Each dot represents a single cell. Salmon pink: AGS, green: NCI-N87, blue: SNU-1, purple: gastric CTCs) (**B**) Heatmap of overexpressed genes of each subgroup. Top 30 overexpressed genes from each subgroup are shown. Heatmap showed log scaled RPM that increases from purple to yellow. **C** Violin plot for expression of EMT-related genes, epithelial genes, platelet related-genes, TGF-β stimulated genes. Each dot represents a single-cell. The red bar represents mean value. The black bar represents deviation.

**Fig. 5:**
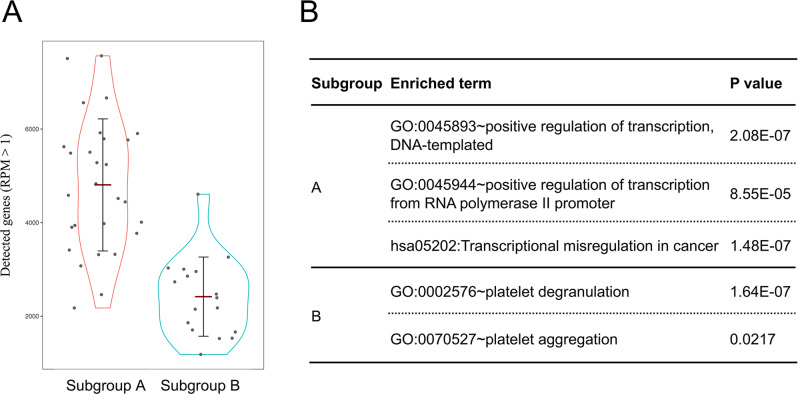
Comparison of subgroup A and B. **A** Comparison of the number of detected genes (RPM > 1) between subgroup A and B. Each dot represents a single-cell. The red bar represents mean value. The black bar represents deviation. **B** Gene ontology analysis of enriched genes.

Next, unsupervised analysis of the transcriptome of single-CTCs was performed. In recent years, unsupervised clustering by transcriptome analysis has enabled the discovery of new cell subpopulations based on expression patterns^48^. We classified CTCs into multiple subtypes, and performed clustering by Seurat, a package for single-cell RNA-seq analysis, and extracted genes that characterize each cluster^49^. Figure 4A is a Uniform Manifold Approximation and Projection (UMAP) plot that illustrates the clustering of CTCs and cancer cell lines. Each dot indicates one cell, G (green) indicates gastric cancer CTCs, and AGS (salmon pink), N87 (blue), and SNU1 (purple) indicates gastric cancer cell lines. Single cells were classified into three subgroups; most of the CTCs belonged to subgroups A and B. The cell lines and only two CTCs belonged to subgroup C. We measured nCount and nFeature of CTCs to confirm that clustering analysis revealed biological differences. We confirmed that there were no remarkable differences in nCount between each subgroup, but the nFeature was larger for subgroups A and C than for subgroup B (Supplementary Fig. 5). CTCs in subgroup B were detected in patients with CTCs in subgroups A and C (Supplementary Table 1). Thus, we conclude that our clustering results did not show technical differences but biological differences in CTCs. Figure 4B is a heat map displaying the characteristic overexpressed genes in each subgroup (names of overexpressed genes was shown in Supplementary Table 2). Purple color indicates low expression level of genes, whereas yellow represents high expression level. Subgroup A was found to express EMT-related transcription factors such as *ZEB2*, *MEF2D*, *GATA1*, and *GATA2*, and transcription-coupling factors such as *KMT2A* (Fig. 5B, C). Interestingly, the expression of *NFKBIA*, an inhibitory protein of NF-κB, was observed. *NFKBIA* expression is induced by activation of NF-κB^50^, suggesting that transcriptional induction by the NF-κB pathway occurs in this subgroup of cells. Subgroup B showed strong expression of genes related to platelet activation and aggregation, such as *ITGA2B*, *PF4* (Platelet Factor 4), and *PPBP* (pro-platelet basic protein, isoform of *CXCL7*) (Fig. 4C). Furthermore, platelet-related genes such as *TGFB1* and *SPARC* were also expressed in subgroup A, suggesting that subgroups A and B are cells to which platelets adhere. In recent years, it is becoming evident that contact between platelets and cancer cells leads to progression of EMT. According to Labelle et al., TGF-β released from platelets activates the SMAD signaling pathway in cancer cells, and NF-κB activates EMT in the cancer cells-platelet interaction^51^. Besides, recent studies have also revealed the importance of the interaction between cancer cells and platelets in EMT^52^. Thus, we inferred that EMT-related gene expression and *NFKIB* expression observed in subgroup A were due to contact with platelets. This observation suggests that gastric CTCs undergo EMT through platelet adhesion. In contrast, expression of EMT-related genes was not detected in subgroup B, and the number of expressed genes (nFeature) tended to be lower than that of subgroup A (subgroup A: 4805 ± 1412 genes; subgroup B: 2418 ± 845 genes) (Fig. 5A). Besides, the expression level of platelet-related genes in subgroup B was higher than that of subgroup A (Fig. 4C). Gene Ontology analysis also showed that subgroup A strongly expressed transcription-related genes (GO:0045893, positive regulation of transcription, DNA-templated; GO:0045944, positive regulation of transcription from RNA polymerase II promoter) (Fig. 5B, Supplementary Fig. 6A and Supplementary Data 2) and subgroup B strongly expressed platelet-related genes (GO: 0002576, platelet degranulation; GO:0070527, platelet aggregation) (Fig. 5B, Supplementary Fig. 6B and Supplementary Data 3). These results suggested that cells in subgroup B had more adhered platelets or had a lower CTC transcriptional activity. Interestingly, the expression of cell cycle regulators such as *CDKN1B* and *CDKN1A*, which are known to be induced by TGF-β, was observed in subgroup A (Fig. 4C). These regulators cause cell cycle arrest in the G1 phase; *p21* is known to contribute to resistance to chemotherapy^53^. Furthermore, subgroup A CTCs were detected in patients who failed to respond to first-line treatment and had a longer duration of therapy before CTC collection. (Supplementary Table 3 and Supplementary Fig. 7). From this result, it was inferred that CTCs in subgroup A were resistant to chemotherapy.

In subgroup C, which includes cancer cell lines, many genes related to metabolic pathways and epithelial markers were detected as overexpressed genes. We concluded that subgroup C comprises of actively metabolizing cells. Platelet-related genes were not expressed in CTCs belonging to subgroup C, and high expression of epithelial markers such as *EpCAM*, *KRT8*, and *KRT18*, absent in subgroups A and B, was detected (Fig. 4C). This suggests that CTCs in subgroup C have completely different characteristics from other CTCs. We note that all patients (*n* = 2) in subgroup C died within 1 month of blood collection (Supplementary Table 1). Therefore, classification of patients into subgroups according to the gene expression pattern of cancer cells may be effective in predicting the prognosis of gastric cancer patients.

## Discussion

In this study, we characterized the CTCs of patients with metastatic gastric cancer by single-cell RNA-seq analysis. There are several reports of the recovery of gastric cancer CTC by the CellSearch System^27^. However, the low detection rate of CTCs and low cut-off value suggest that there are numerous CTCs that cannot be detected due to EMT^28^. Recently, clinical studies revealed the advantage of a size-selective CTC enrichment method for the detection of gastric CTCs^54,55^. Thus, the size-selective method has the potential to achieve higher sensitivity in CTC detection from metastatic gastric cancer patients than the CellSearch system. In particular, Cheng and colleagues showed CTCs with mesenchymal gene (vimentin and twist) expression by RNA-in situ hybridization (RNA-ISH) and correlated the number of mesenchymal-CTCs with disease progression^30^. Although RNA-ISH is powerful tool for identifying the EMT-status of CTCs, it is difficult to obtain information about the molecular mechanism of EMT progression because of the small number of marker genes. The size-selective CTC enrichment method has great potential for understanding of the mechanism of EMT in gastric CTCs. Therefore, we adopted the size-selective CTC enrichment method and the surface antigen-independent CTC detection method (CellTracker Green-positive and CD45-negative). As a result, CTC candidates were detected in 96% (26/27) of the cases. The detection rate of CTC in gastric cancer was remarkably high compared to previous reports (11–85%), and the size-selective CTC recovery method was found to be effective for the detection of gastric cancer CTCs.

About half of the obtained CTCs were low-quality cells in which no amplification product was obtained or no housekeeping gene expression was observed. The QC pass rate was much lower than that of cancer cell lines. These results suggested that the RNA quality of single-CTCs were found to be lower than that of cancer cell lines. It is reported that cytoplasmic mRNA are degraded as an early part of the apoptotic response^56^. Thus, CTCs with low-quality RNA may be in the early phase of apoptosis owing to several factors such as shear stress in blood vessels^57,58^. Previous studies with single CTCs from breast cancer and prostate cancer have reported analysis issues with 30–70% of CTCs due to poor quality^7,18,59^. The QC pass rate obtained in this study was comparable to previous findings, suggesting that it is a common property among all cancer types.

We expected that the detection of CD45-negative cells as CTCs will be influenced by contamination of hemocyte cells, however, this effect can be negated by identifying the expression of hemocyte-related genes. All CTC-candidates in the present study were of a different species from PBMC as shown by unsupervised clustering. Till date, most single-cell RNA-seq analyses of CTCs utilize antibody staining of epithelial markers such as EpCAM and E-cadherin to detect CTC, and many isolated CTCs exhibit epithelial gene expression^7,12,18^. Conversely, most of the CTCs detected in this study were cells that lost epithelial gene expression due to EMT. Thus, isolation of CD45-negative cells as CTC candidates allows the inclusion of EMT-induced CTCs.

The majority of CTCs expressed genes associated with platelet binding. As platelet-specific genes such as *PF4* and *PPBP* were expressed, we assumed that the platelets were attached to the separated CTCs. In recent years, it has become clear that the adherence between cancer cells and platelets not only protects cancer cells from immune cells and shear stress, but also promotes EMT. Previous studies have reported cases in which platelet adhesion was suggested in CTCs isolated from mice model of pancreatic cancer^12^. It has been reported that CTCs or circulating tumor microemboli (CTM) with attached platelets were detected in human samples, but gene expression analysis of these cells was not performed^60^. In the present study, CTCs with predicted platelet adhesion did not express epithelial marker genes. This result shows that it is possible to detect CTCs independent of epithelial markers. Besides, a strong expression of *TGFB1* was observed in CTCs with advanced EMT. TGF-β is mainly secreted by platelets and induces EMT by activating the SMAD pathway in cancer cells^51^. In recent years, it has been revealed that binding of cancer cells to platelets promotes the production of TGF-β by cancer cells and synergizes EMT with platelet-derived TGF-β^61^. Taken together, we propose that platelet adhesion plays a major role in the induction of EMT in CTCs. Recent study detected CTM from 24% of patients with stage IV gastric cancer before chemotherapy^62^. Abdallah et al. reported decreasing of CTM detection rate in local gastric cancer patient after the neoadjuvant chemotherapy and surgery^63^. These studies implicate that the chemotherapy might decrease CTMs. We did not detect CTM from our cohort including patient without chemotherapy. However, our previous study demonstrated the detection of CTM in metastatic non-small cell lung cancer patients by MCA system^64^. Thus further study will reveal the relationship between EMT and platelet-CTM interaction in gastric cancer patients.

Recently, Sun et al. have shown that hepatocellular carcinoma CTCs possess epithelial properties immediately after they are released from tissues, but EMT may induced within pulmonary capillaries. Also, Aceto et al. have shown that the TGF-β pathway activity of CTC is high in cases of metastasis to other organs in breast cancer. Based on these findings, it was suggested that CTC activate the TGF-β/SMAD pathway and induces EMT by adhesion to platelets in blood vessels. Generally, platelet-cancer cell interaction is analyzed by in vitro experiments or mouse models. Our results showed that single-cell analysis of CTC may reveal more information about the function of platelets in metastasis and may contribute to the development of new therapeutic targets. Recent studies have highlighted the importance of tumor-educated platelets in cancer progression^52^. Our results suggest that this single CTC isolation system could contribute to revealing the direct communication between CTCs and platelets. Moreover, the expression of *CDKN1A* (p21) and *CDKN1B* (p27) was observed in most EMT-induced CTCs, suggesting that the cell cycle is arrested in the G1 phase. These genes are known to be involved in chemoresistance of cancer cells and dormancy. Therefore, gene expression analysis of CTC may be effective in analyzing the mechanism of acquisition of resistance to chemotherapy in gastric cancer, and may aid decisions involving treatment selection for patients.

The clinical cases in which CTCs exhibited epithelial gene expression (G_12, G_25) had a considerably worse prognosis than other cases. The epithelial CTCs showed a similar gene expression pattern to the cell lines, with a strong expression of genes associated with various metabolic pathways. This suggests that these CTCs are more metabolically active than other CTCs. Among cases of metastatic breast cancer with extremely high number of epithelial CTCs, death has been reported within 1 month of blood collection^1,65,66^. It was also confirmed that the number of epithelial CTCs was significantly high in patients who did not show response to therapeutics in cases of metastatic gastric cancer^67^. Furthermore, single-cell gene expression analysis of CTCs in pancreatic cancer cases also report a poor prognosis in cases wherein CTCs show epithelial characteristics^59^. Taken together, these findings suggest that CTCs with epithelial gene expression may be an indicator of poor prognosis. The CTC detection method using the CellSearch system or immunostaining detects CTCs based on the expression of an epithelial protein, however, it is difficult to quantitatively evaluate the expression level. As single-cell gene expression analysis facilitates a more quantitative characterization of CTC, it may be used for the pursuit of new prognostic markers. Moreover, no expression of platelet adhesion-related genes was observed in epithelial CTCs. Therefore, we propose that these CTCs indeed avoid the immune system and fluid shear caused by blood flow. However, a conclusion cannot be drawn on the exact mechanism of this function of CTCs and further analysis is required in this direction. In addition, our analysis involved a limited cohort size and a small number of baseline patients; a larger scale analysis is warranted in future. We plan to perform a large-scale clinical study for validation of marker genes extracted in this study. Furthermore, we noted that the size of gastric CTCs tended to be smaller than that of cancer cell lines. This observation suggests that there might be small CTCs that are difficult to entrap using the size-selective method. Further optimization for size-selective CTC enrichment process is still required.

In summary, we constructed a pipeline for size-selective and antigen-independent CTC separation and single-cell RNA-seq analysis based on the MCA/GCM method. A majority of the gastric cancer CTCs were EMT-induced cells, and we suggest that the adhesion of platelets initiates a signal transduction, which leads to the induction of EMT, a phenomenon mainly occurring in blood vessels. Furthermore, our results suggest that CTCs exhibit cell cycle arrest and are associated with resistance to chemotherapy. Furthermore, some CTCs were epithelial and were found to be useful markers for prognosis prediction.

## Methods

### Cell lines and culture

Human lung cancer cell lines, NCI-H1975 (ATCC^®^ CRL5908^™^) and PC9 (ECACC 90071810), and human gastric cancer cell lines, AGS (ATCC^®^ CRL-1739^™^), SNU-1 (ATCC^®^ CRL-5971^™^), and NCI-N87(ATCC^®^ CRL-5822^™^) were used in this study. The cell lines were cultured in RPMI-1640 media containing 10% (v/v) fetal bovine serum (Invitrogen Corp., Carlsbad, CA) and 1% (v/v) penicillin/streptomycin (Invitrogen Corp.) for 3–4 days at 37 °C with 5% CO_2_ supplementation. Prior to experiments, all cells, except for SNU-1 (floating cells), grown up to 80% confluency were trypsinized and re-suspended in PBS. SNU-1 cells were directly recovered from the media and re-suspended in PBS. Cells were then stained with 5 μM CellTracker Green 5-chloromethylfluorescein diacetate (CMFDA; Thermo Fisher Scientific, Waltham, MA) and 1 μg/ml Hoechst 33342 (Thermo Fisher Scientific) for 20 min and centrifuged at 400 *g* for 3 min to obtain cell pellets. After washing the cells twice with PBS, they were re-suspended in PBS containing 2 mM EDTA and 0.5% bovine serum albumin (BSA; PBS-EB). For spike-in experiments, SNU-1 (100 cells) was spiked into human blood (1 mL). Human blood samples were collected from healthy donors at the Tokyo University of Agriculture and Technology. Experimental protocols were approved by the Institutional Review Board of the Tokyo University of Agriculture and Technology (Approval code: No. 30-10).

### Patient samples

The clinical study was conducted in Tokyo Metropolitan Cancer and Infectious Diseases Center, Komagome Hospital. The study design involving human subjects was approved by the Institutional Review Board at Tokyo Metropolitan Cancer and Infectious Diseases Center Komagome Hospital (Approval Number: 1441). Blood samples were collected from patients with metastatic gastric cancer treated with chemotherapy (*n* = 24) from April 2017 to October 2019. We also obtained blood samples from three metastatic gastric cancer patients without any chemotherapy. Detailed disease status and therapy are provided in Table 1. Written informed consent was obtained from all patients prior to blood sampling. For each patient, blood sample was collected by venipuncture into Venoject II 5 ml EDTA-2Na tubes (VP-NA050KN, Terumo, Japan). Peripheral blood samples for CTC analysis were collected after withdrawal of the first several milliliters of blood for clinical use to avoid potential skin cell contamination from the venipuncture. All the blood samples were processed within 2 h of collection.

**Table 1:** Summary of patients.

Patient ID	Sex	Age	Metastasis	Treatment at time of CTC collection	Response	Prior treatment
G1	Man	66	Liver	SOX	PD	Trastumab + FP, SOX
G2	Woman	68	Liver	No	N/A	TS-1
G3	Man	75	Liver, Lymph node	No	N/A	TS-1
G4	Woman	81	Peritoneal dissemination	No	N/A	None
G5	Woman	63	Lung, Pancreas	PTX	PD	SOX
G6	Woman	60	Esophagus	No	N/A	None
G7	Woman	60	Esophagus	No	N/A	None
G8	Man	73	Peritoneal dissemination	No	N/A	S-1
G9	Man	65	Peritoneal dissemination, Lymph node	SOX	Response	SOX
G10	Man	58	Peritoneal dissemination, Lymph node, Liver	SOX	Response	SOX
G11	Woman	52	Peritoneal dissemination	RAM + PTX	PD	SOX, RAM + PTX
G12	Man	71	Peritoneal dissemination	No	N/A	FOLFOX, nab-PTX
G13	Man	69	Peritoneal dissemination, Lymph node, Liver	FOLFOX + BV	PD	FOLFOX + BV
G14	Man	76	Paraaortic lymph node, Liver	SOX	Response	SOX
G15	Man	71	Peritoneal dissemination, Paraaortic lymph node, Pancreas	SOX	Response	SOX
G16	Woman	68	Liver	CPT-11	Response	TS-1, PTX + RAM, Nivolmab, CPT-11
G17	Man	75	Liver, Lymph node	No	N/A	TS-1, PTX + RAM, Nivolmab, CPT-11
G18	Woman	68	Liver	No	N/A	TS-1, PTX + RAM, Nivolmab, CPT-11
G19	Man	72	Liver	5-FU + Tmab	Response	5-FU + Trastumab
G20	Woman	60	Peritoneal dissemination	FOLFOX	Response	FOLFOX
G21	Woman	68	Liver	No	N/A	TS-1, PTX + RAM, Nivolmab, CPT-11
G22	Woman	81	Peritoneal dissemination	No	N/A	SOX, PTX + RAM
G23	Man	70	Peritoneal dissemination	No	N/A	FOLFOX, RAN + nab-PTX, Nivolmab
G24	Man	75	Peritoneal dissemination	RAM + nabPTX	Response	S-1, RAM + nab-PTX
G25	Man	81	Lymph node	SOX	Response	SOX
G26	Man	66	Peritoneal dissemination	SOX	Response	FOLFOX, SOX
G27	Man	78	Peritoneal dissemination, Colon	No	N/A	SOX

### Single-cell isolation by micromanipulation

Prior to micromanipulation-based single-cell isolation, a spacer seal (a slide seal for in situ PCR, inner space: 9 × 9 mm^2^, thickness: 300 μm, Thermo Fisher Scientific, Waltham, MA, USA) was affixed at the glass slide (Matsunami Glass, Osaka, Japan). Cellular RNA was stabilized using CellCover (AL Anacyte Laboratories UG, Hamburg, Germany), if required, according to the manufacturer’s protocol. After determination of cell concentration by a hemocytometer, ~100 cancer cells were suspended in 100 μl of PBS-EB. The cell suspension was mounted only on the inside of the spacer seal and observed under a fluorescence microscope (IX71; Olympus Co., Tokyo, Japan). Single cancer cells exhibiting strong florescence were manually picked up by a micromanipulator equipped with a 30 μm diameter glass capillary (PicoPipet; Nepa Gene Co., Ltd., Chiba, Japan). Then, single cells were transferred into 0.4 μL of a lysis buffer (0.5% NP40) in 200 μL PCR tubes (N8010840, Thermo Fisher Scientific). In this study, we use 0.5% NP40 as lysis buffer for subsequent Quartz-seq based whole transcriptome amplification. Isolated single-cells were immediately frozen with liquid nitrogen and stored at −80 °C for further whole transcriptome amplification.

### Fabrication of CTC recovery device

An MCA made of nickel by electroforming (Optonics Precision Co. Ltd.) was used for CTC recovery. The circular pore with a diameter of 8 μm was fabricated for entrapment of the CTCs. The pore diameter was previously optimized for size- and deformability-based CTC enrichment^31^. The distance between pores was 125 μm, with a total of 3969 cavities arranged in each 63 × 63 array for subsequent GCM steps. The CTC recovery device was fabricated by integration of MCA, PMMA-made mold and PDMS-made flow channel using spacer tape^31^.

### CTC enrichment and staining

One ml of cancer cell-spiked PBS-EB (or blood) or patient blood was introduced into the PBS-EB pre-filled CTC recovery device. Subsequently, negative pressure was applied to the samples with a peristaltic pump (MINIPULS 3; Gilson, Wisconsin, USA) which was connected to a vacuum line. The sample was passed through the MCA at a flow rate of 150 μl/min. To remove any residual cells, 2 mL of PBS-EB was passed through the MCA at a flow rate of 200 μL/min for 10 min. For the patient blood samples, 1 mL PBS-EB containing 5 μM CellTracker Green CMFDA and 1 μg/ml Hoechst 33342 was introduced into the MCA and incubated for 20 min and the excess dye was washed with 1 mL PBS-EB. Then, RNA of recovered cells was stabilized with CellCover throughout the MCA for 15 min. After washing with 1 mL PBS-EB, cancer cell-spiked PBS-EB samples were moved to the GCM step. For patient blood samples, cells were stained with anti-CD45 antibody (Hitachi Chemical, Tokyo, Japan) and Texas Red-labeled secondary antibody (Hitachi Chemical). Finally, the array was washed with 1 mL of PBS-EB and PBS to remove excess antibodies.

### Single-cell isolation based on GCM

Prior to GCM, polyethylene glycol diacrylate (PEGDA, Mn = 700; Sigma-Aldrich) with 0.5% Irgacure 2959 (photoinitiator) was introduced onto the MCA. Then, the CTC recovery device was covered with a coverslip (24 × 40 mm; Matsunami Glass). Coverslips were modified with 3-(trimethoxysilyl)-propyl methacrylate to provide a stiff connection between the coverslip and hydrogel. The MCA was observed under a fluorescence microscope (BX53; Olympus Co.), and cancer cells and CTC-candidates were determined as CellTracker Green- and Hoechst-33342 positive and CD45-negative cells. The subsequent steps were performed as previous study as follow^32^. Briefly, single-cells were encapsulated into the hydrogel by light irradiation (λ = 365 nm, 12.7 mW/cm^2^ for 30 s). After peeling off the coverslip, hydrogel-encapsulated single-cells were resuspended into 0.4 μl of lysis buffer (0.5% NP40) in 200 μL PCR tubes (N8010840, Thermo Fisher Scientific) using tweezers. Isolated single-cells were immediately frozen with liquid nitrogen and stored at −80 °C for further whole transcriptome amplification.

### Single-cell whole transcriptome amplification

A recent study revealed the superior performance of poly A tailing-based WTA in detection of genes^68^. In addition, there are several reports of single-cell transcriptome analysis of CTCs using poly A tailing-based WTA^7,12,14,15^. Thus, we selected Quartz-seq, based on the poly A tailing reaction, for single-cell WTA in the present study^37^. In brief, single cells in lysis buffer were placed in an aluminum PCR rack on ice and subsequently mixed with 0.8 μL primming buffer (1.5× PCR buffer with MgCl2 [TaKaRa Bio], 41.67 pmol/L of RT primer, 4 U/μL of RNase inhibitor [RNasin Plus; Promega Corp., Madison. WI, USA], and 50 μmol/L dNTPs). After mixing and centrifuging (3000 × *g*, 10 s), the mRNA from single cells was denatured and captured by RT primer, oligo dT linked to an adaptor sequence, using a thermal cycler (70 °C for 90 s and 35 °C for 15 s). Reaction mixtures were placed in an aluminum PCR rack on ice, and treated with 0.8 μL of RT buffer (1× PCR buffer, 25 U/μL reverse transcriptase [SuperScript III; Life Technologies], and 12.5 mmol/L DTT). First-strand cDNA was synthesized using a reverse-transcription reaction (35 °C for 5 min and 45 °C for 20 min). After heat inactivation at 70 °C for 10 min, the reaction tubes were placed in an aluminum PCR rack on ice and treated with 1 μL of the exonuclease solution (1× Exonuclease buffer [TaKaRa Bio] and 1.5 U/μL exonuclease I [TaKaRa Bio]). The primer digestion reaction was performed to remove excess primers (37 °C). Reaction tubes were placed in an aluminum PCR rack on ice after heat inactivation at 80 °C for 10 min, and subsequently treated with 2.5 μL of poly-A-tailing buffer (1 × PCR buffer, 3 mmol/L dATP, 33.6 U/μL terminal transferase [Roche Applied Science], and 0.048 U/μL RNase H [Invitrogen]). The poly A tailing reaction was performed at 37 °C for 50 s and heat inactivated at 65 °C for 10 min. The reaction samples were placed in an aluminum PCR rack on ice and mixed with 23 μL of the second strand buffer (1.09× MightyAmp Buffer v2 [TaKaRa] and 0.054 U/μL MightyAmp DNA polymerase [TaKaRa]). The second-strand cDNA reaction was performed (98 °C for 130 s, 40 °C for 1 min, and 68 °C for 5 min), and reaction tubes were placed in an aluminum PCR rack on ice. Then, 25 μL of PCR buffer (1× MightyAmp Buffer v2 and 1.9 mol suppression PCR primer) was immediately added, and PCR was performed under the following conditions: 98 °C for 10 s, 65 °C for 15 s, and 68 °C for 5 min (21 cycles). Amplified cDNA was purified by the MinElute PCR Purification Kit (QIAGEN, Hilden, Germany) and eluted in 24 μl of buffer EB. Then, yield and average length of amplified cDNA were measured using the High Sensitivity DNA Kit (Agilent, California, USA). For quality checks, amplification of *GAPDH* by qPCR using 50 pg of amplified cDNA as template was performed. Only cells with detectable levels of *GAPDH* were subjected to further qPCR and selected for library construction for RNA-seq analysis.

### RT-qPCR of WTA products

WTA products (50 pg) were subjected to qPCR analysis using MightyAmp^TM^ for Real Time (TB Green Plus) kit (TaKaRa Bio, Shiga, Japan). Briefly, 5 μl of diluted WTA products were mixed with 0.8 μl of primers (10 μM of each primer), 10 μl of MightyAmp for Real Time (TB Green Plus) master mix (2X), 0.4 μl of ROX reference dye II (50X), and 3 μl of ultrapure water. The thermal cycler was set to carry out PCR under the following conditions: 98 °C for 1 min; 40 cycles of 98 °C for 10 s and 60 °C for 20 s min; 60 °C for 30 s and Cq values were measured. Eleven genes were selected from PrimerArray^®^ Small Cell Lung Cancer & Non-small Cell Lung Cancer (Human) (TaKaRa) kit for evaluation of amplification bias. The expression pattern of 96 genes in total RNA enriched from NCI-H1975 cells were evaluated according to the manufacturer’s protocol. Then, we selected *GAPDH* and *ACTB* as high-expression genes, *TP53* and *GUSB*, *PIAS3*, and *CDK2B* as intermediate-expression genes and *KRAS*, *CLO4A2*, *AKT3*, *LAMA2*, and *CYCS* as low-expression genes. All the primers were re-designed using PrimerBLAST to amplify regions <150 bp within the 250 bp stretch from the 3′ end of mRNA (Supplementary Table 4) for polyA-based WTA products. The specificity of amplification of each RT-qPCR was evaluated by melting-curve analysis.

### RNA-seq

Ten nano grams of amplified cDNA was used for library construction with NEBNext Ultra DNA Library Prep Kit for Illumina (New England BioLabs, Massachusetts, USA). The cDNA was sheared by the Covaris system^69^, and then the sheared fragments were end-repaired, A-tailed, and ligated to sequencing adaptors according to manufacturer’s protocol. A size selection of about 200 bp was performed before the PCR enrichment (12 cycles). Concentration of the cDNA library was quantified using a Qubit 2.0 fluorometer (Thermo Fisher), and diluted to 2 ng/μl before checking the insert size on an Agilent 2100 and quantifying by qPCR for greater accuracy (library activity > 2 nM). Libraries were subjected to Illumina Hiseq 2500 and 50-base single-ended reads to a depth of 5 M reads per samples were obtained. These experimental procedures were performed by Novogene Corporation.

### Analysis of RNA-seq data

All raw RNA-seq reads were subjected to trimmomatic-0.36-5 to remove the sequencing adaptors and WTA primers. Trimmed reads were aligned to the human reference genome (hg19) using HISAT2 2.1.0 with default parameters. Aligned reads per genes were counted using HTseq. Transcriptomic reads were aligned using a GTF file with transcript annotations obtained from GENCODE (release 19, ftp://ftp.ebi.ac.uk/pub/databases/gencode/Gencode_human/release_19/gencode.v19.annotation.gtf.gz). The read counts were divided by the total number of reads and multiplied by one million to form the reads-per-million (rpm). We used rpm rather than rpkm because 3′ bias in the alignments was observed as described in a previous study^12^. Gene body coverage was calculated using RSeQC-2.6.5 for RefSeq registered 63,169 transcripts. In addition, we excluded single cells with a high mitochondrial gene ratio (>25%) from further gene expression analysis according to a previous study^68^. Visualization of gene expression data was performed with R software. Heatmap of known target genes was drawn by heatmap.2 package. Unsupervised hierarchical clustering based on the shared nearest neighbor method was performed by Seurat package^49^.

### Statistics and reproducibility

All statistical analysis except for RNA-seq data analysis were performed by Welch’s *t*-test (two tailed) with an alpha level of 0.05. The statistical analysis were performed using stats v3.6.2 package on R software. In RNA-seq analysis, the gene expression was log-normalized for principal component analysis (PCA) and UMAP methods. For gene ontology enrichment analysis, the significantly differential genes (*p* < 0.05) enriched by Seurat were applied to DAVID^70,71^. All *N* values defined in the legends and manuscript refer to biological replicates unless otherwise indicated.

### Reporting summary

Further information on research design is available in the Nature Research Reporting Summary linked to this article.

## Supplementary information

Supplementary Information

Description of Additional Supplementary Files

Supplementary Data 1-14

Reporting Summary

## Data Availability

All data supporting the findings of this study are available within this article, Supplementary Information and Supplementary Data. We provided an original source data for Fig. 1B, C as Supplementary Data 4 and 5. Source data for Fig. 3A, B as Supplementary Data 6 and 7. Source data for Fig. 4B, C and Supplementary Fig. 6 as Supplementary Data 8. Source data for 5 A as Supplementary Data 9. Source data for Supplementary Figs. 1–5 as Supplementary Data 10–14. Single-cell RNA-seq data were deposited at DRA011720.
