# Supplementary material for: Transcriptomic profiling of single circulating tumor cells provides insight into human metastatic gastric cancer
Source: Commun Biol. 2022 Jan 11;5:20. doi: 10.1038/s42003-021-02937-x (PMC8752828; doi:10.1038/s42003-021-02937-x)
Supplement: Supplementary file 1 — Supplementary Information [file 42003_2021_2937_MOESM1_ESM.pdf]

Transcriptomic profiling of single circulating tumor cells provides insight into human metastatic gastric cancer

Ryo Negishi[1], Hitomi Yamakawa[1], Takeru Kobayashi[1], Mayuko Horikawa[1], Tatsu Shimoyama[2], Fumiaki Koizumi[3], Takeshi Sawada[3], Keisuke Oboki[4], Yasushi Omuro[2], Chikako Funasaka[2], Akihiko Kageyama[2], Yusuke Kanemasa[2], Tsuyoshi Tanaka[1], Tadashi Matsunaga[1] & Tomoko Yoshino[1]

1. Division of Biotechnology and Life science, Institute of Engineering, Tokyo University of Agriculture and Technology, 2-24-16, Naka-cho, Koganei, Tokyo, 184-8588, Japan

2. Department of Chemotherapy, Tokyo Metropolitan Cancer and Infectious Diseases Center Komagome Hospital, Tokyo, Japan.

3. Department of Laboratory Medicine, Tokyo Metropolitan Cancer and Infectious Diseases Center Komagome Hospital, Tokyo, Japan.

4. Center for Medical Research Cooperation, Tokyo Metropolitan Institute of Medical Science, Setagaya-ku, Japan

*Corresponding author. Fax: +81-42-385-7713. Phone: +81-42-388-7021.

E-mail: y-tomoko@cc.tuat.ac.jp

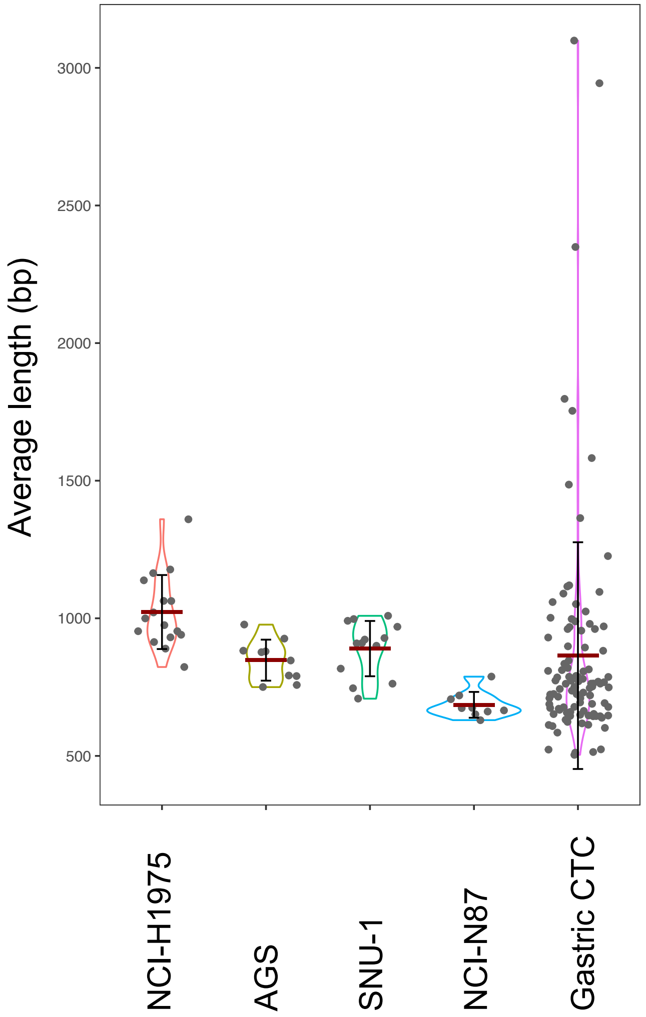

Supplementary Figure 1 Evaluation of average length of WTA products from single cells.

Each dot represents a single-cell. The red bar represents mean value. The black bar represents deviation.

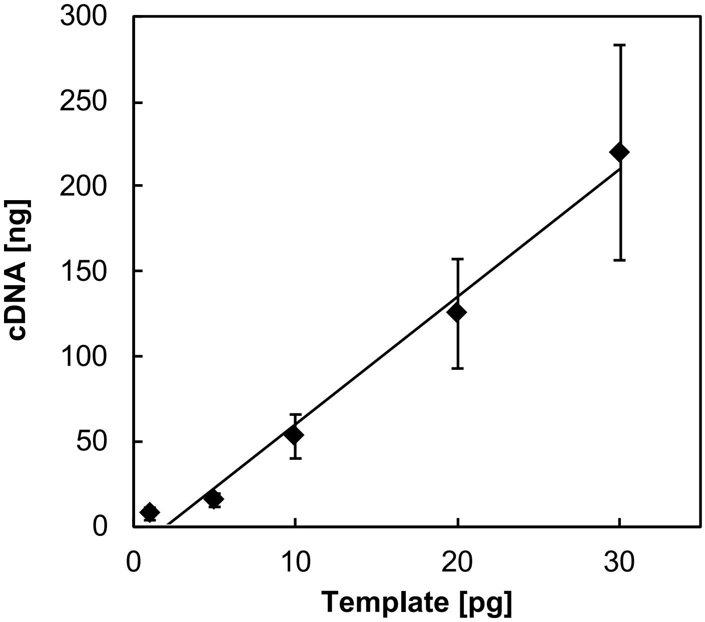

Supplementary Figure 2 Relationship between the amount of input RNA and the yield of amplified product.

Total RNA from NCI-H1975 was subjected to whole transcriptome amplification by Quartz-seq. Error bar shows standard deviation (n = 3).

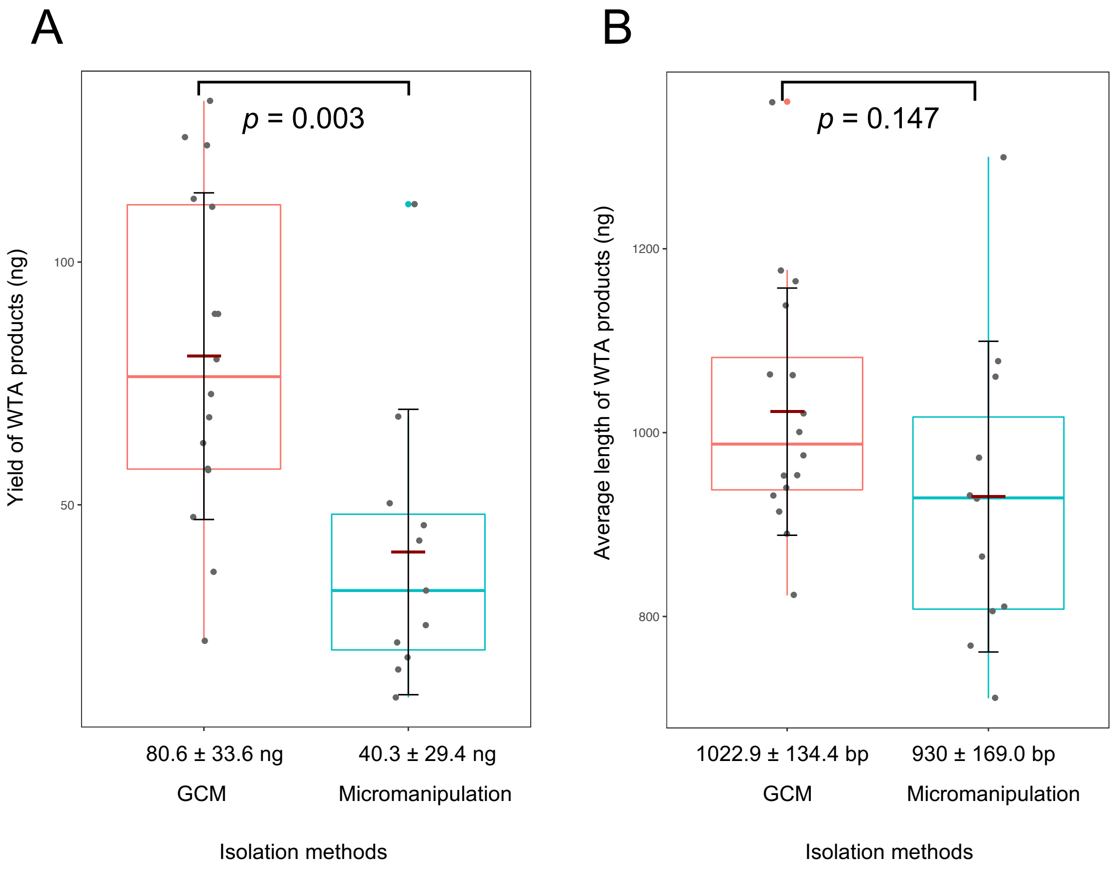

Supplementary Figure 3 Evaluation of amplified cDNA from a single NCI-H1975 cell.

Single-cell isolation and WTA was carried out for NCI-H1975 from the same lot. n=11 biologically independent samples for micromanipulation and n=16 biologically independent samples for GCM. Each dot represents a single-cell. The red bar represents mean value. The black bar represents deviation.

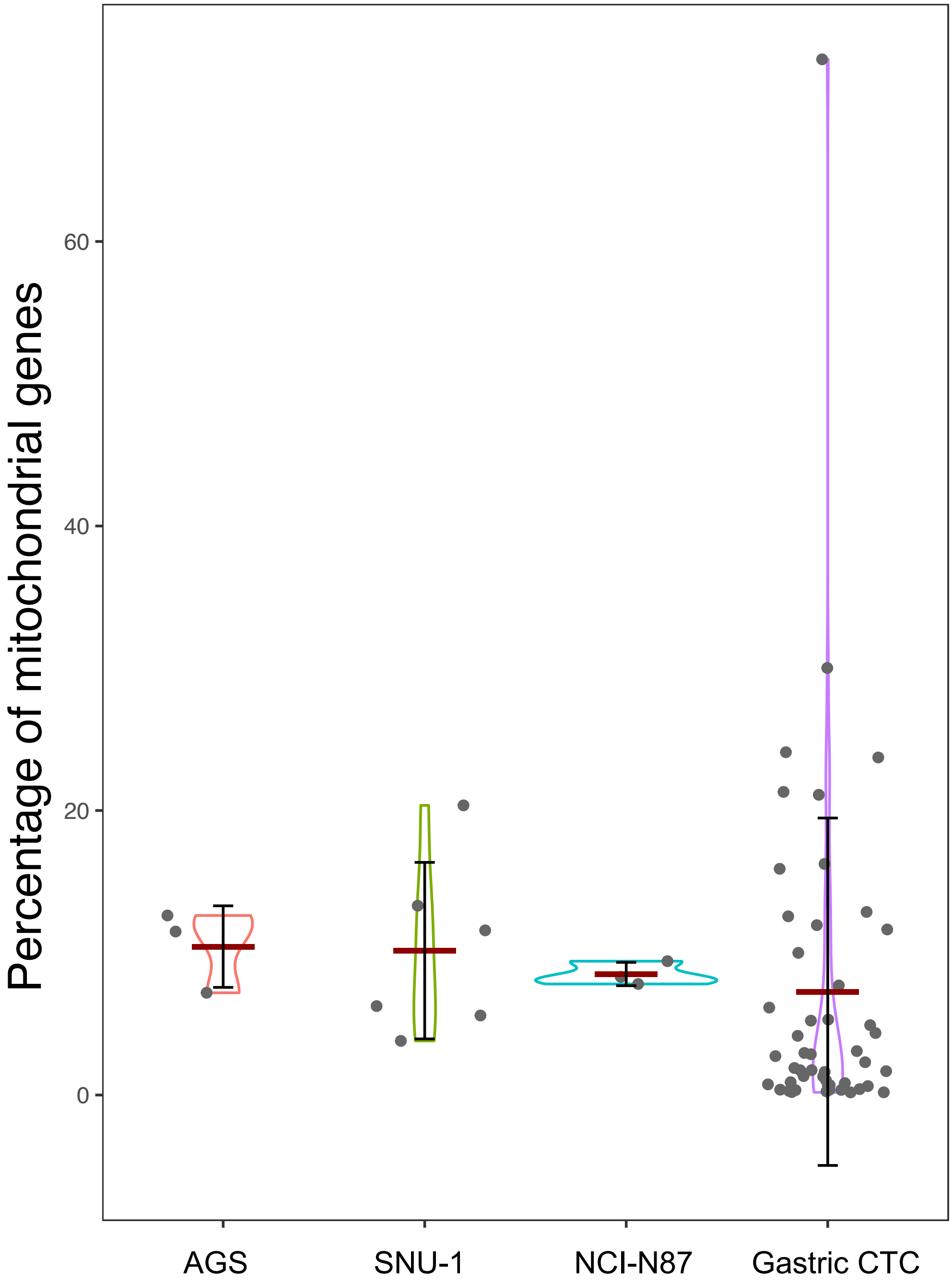

Supplementary Figure 4 Comparison of the mitochondrial genes rate in single-cell sequence library.

Each dot represents a single-cell. The red bar represents mean value. The black bar represents deviation.

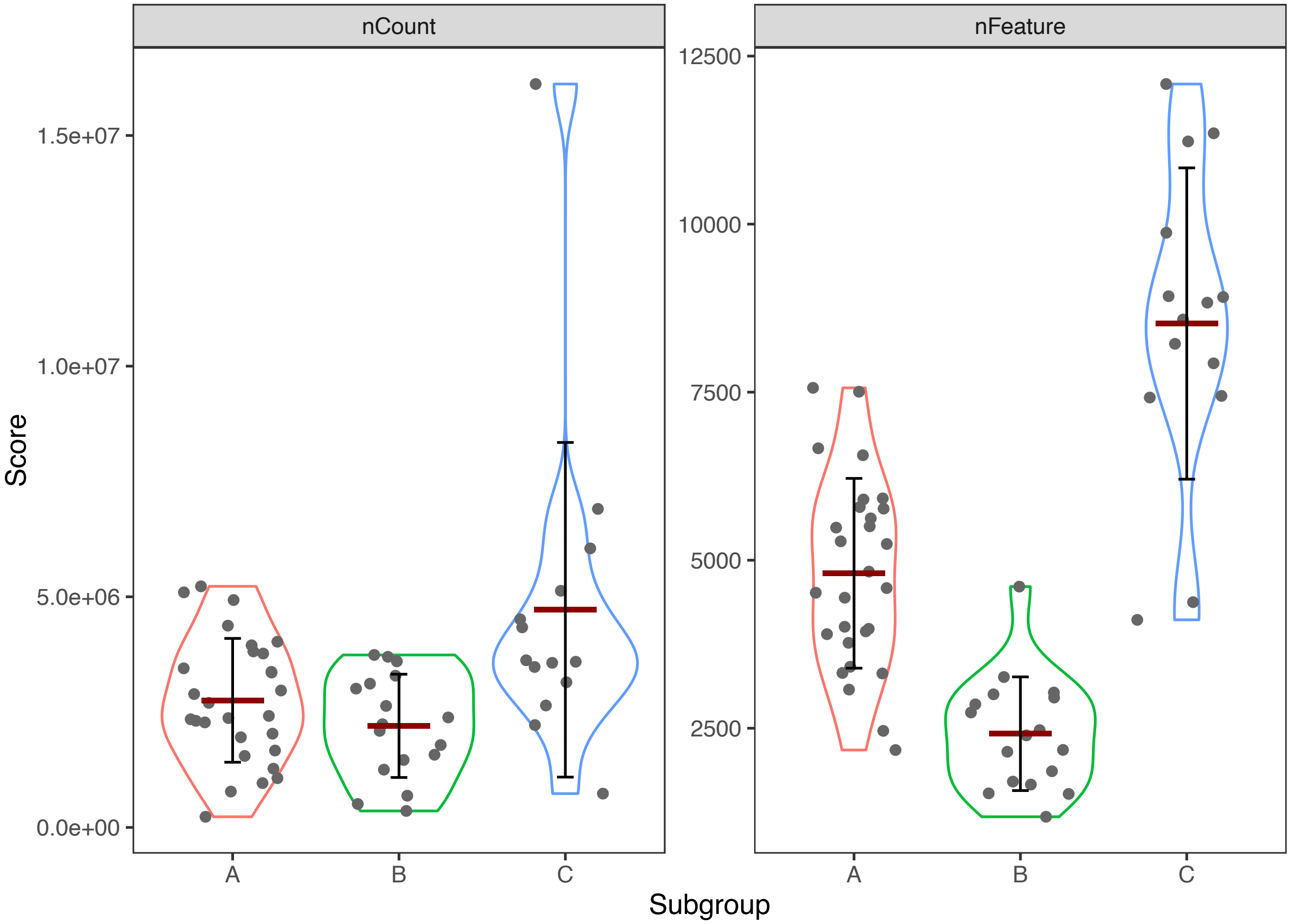

Supplementary Figure 5 Comparison of nCount and nFeature between subgroups. Each dot represents a single-cell. The red bar represents mean value. The black bar represents deviation.

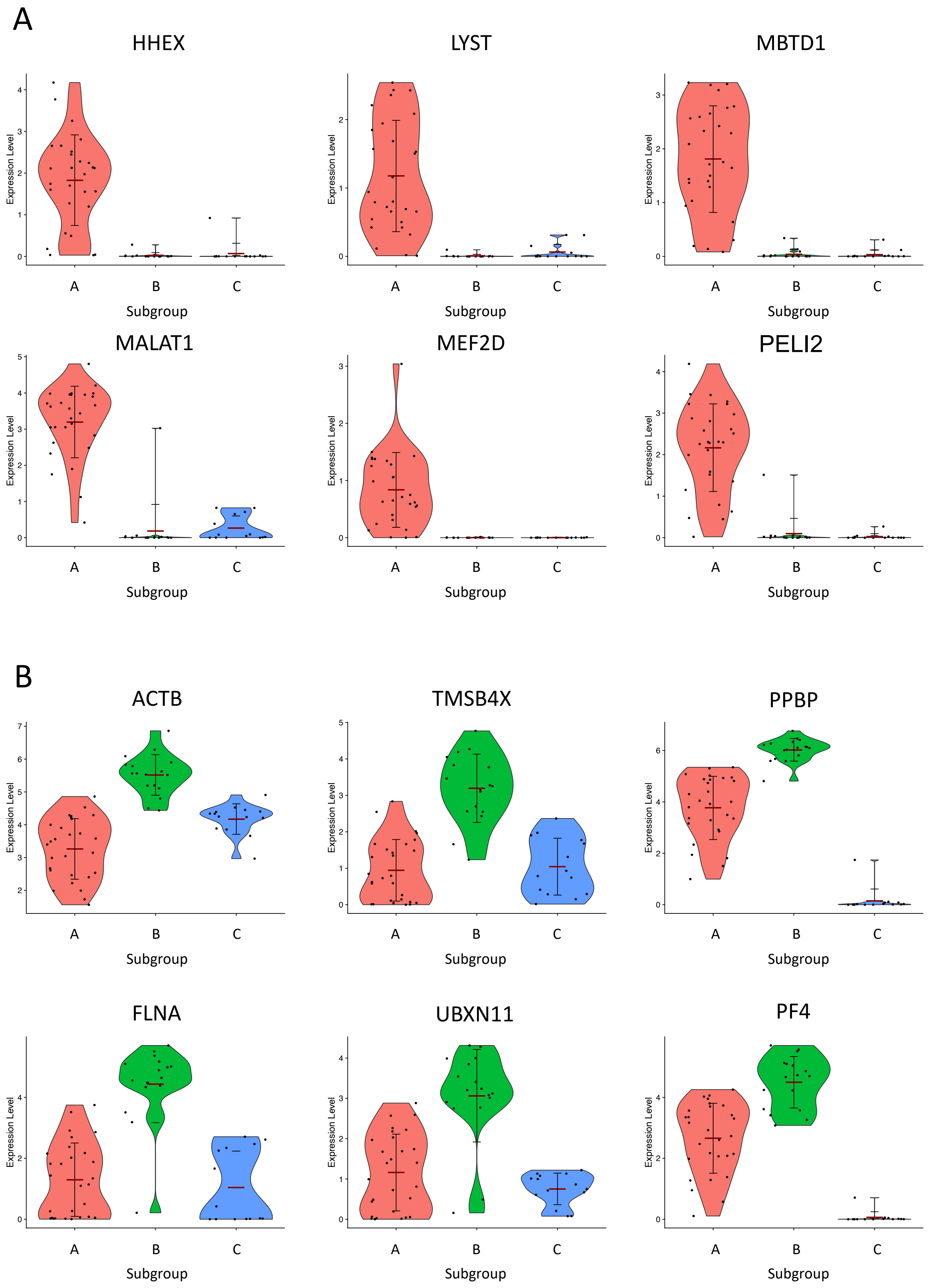

Supplementary Figure 6 Comparison of expression level of genes enriched by GO analysis.
Each dot represents a single-cell. The red bar represents mean value. The black bar represents deviation.

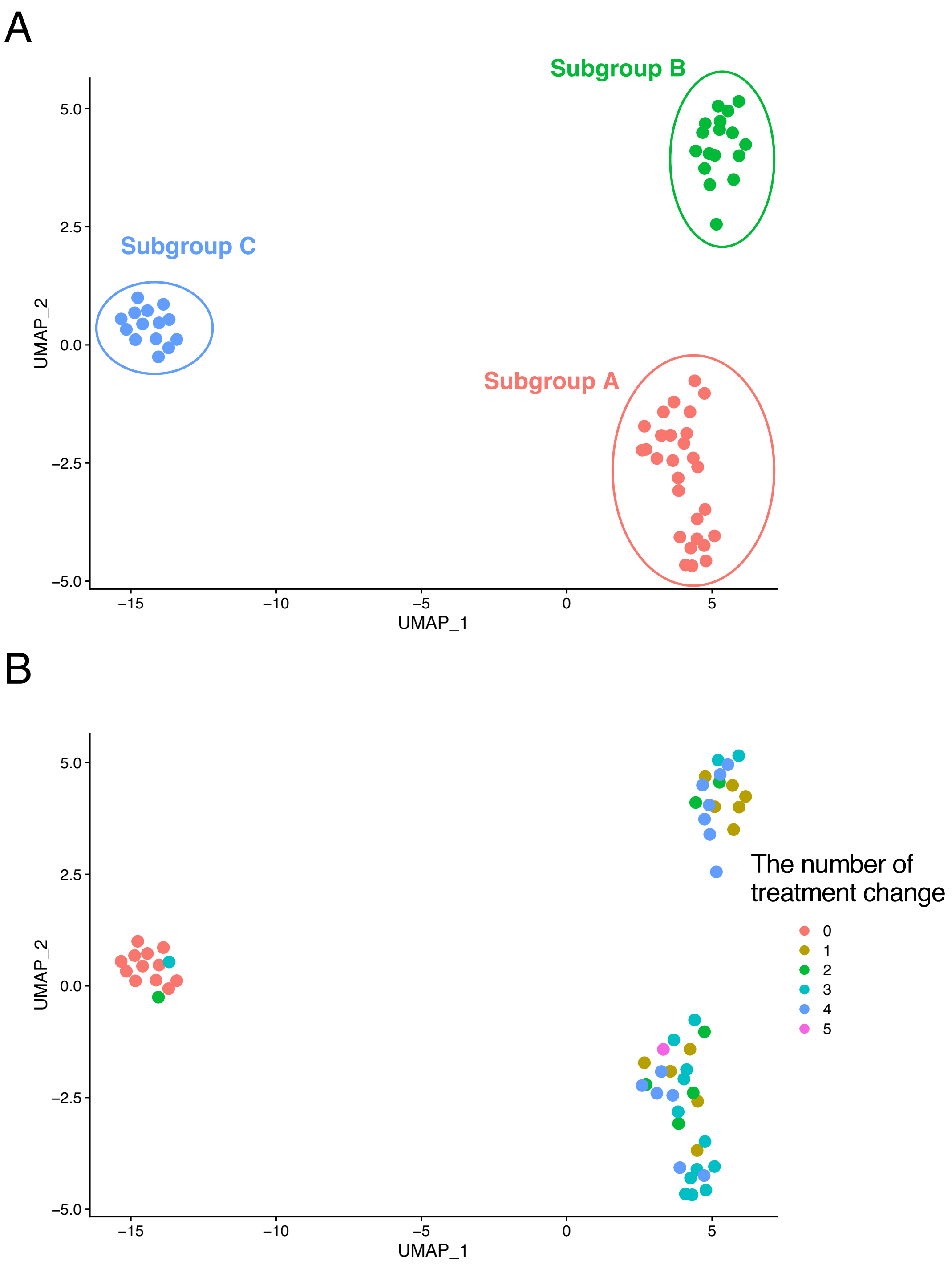

Supplementary Figure 7 Single-cell UMAP plot showing the number of treatment change.

A. Subgrouping (Same as Fig. 5A). B. The number of treatment change.

Supplementary Table 1 Classification of CTCs from gastric cancer patients and overall survival after CTC collection.

Patient	Subgroup A	Subgroup B	Subgroup C	Overall survival after CTC collection	Live/Death
G1	N/A	N/A	N/A	47	Death
G2	1	0	0	880	Live
G3	0	3	0	583	Death
G4	0	1	0	688	Death
G5	N/A	N/A	N/A	148	Death
G6	2	0	0	810	Live
G7	1	1	0	807	Live
G8	1	1	0	612	Live
G9	0	1	0	349	Death
G10	1	0	0	270	Death
G11	1	0	0	261	Death
G12	1	0	1	27	Death
G13	1	1	0	359	Death
G14	0	1	0	540	Live
G15	0	1	0	137	Death
G16	N/A	N/A	N/A	513	Live
G17	2	1	0	148	Death
G18	3	3	0	396	Live
G19	1	1	0	346	Live
G20	0	2	0	341	Live
G21	N/A	N/A	N/A	340	Live
G22	1	0	0	94	Death
G23	10	0	1	10	Death
G24	N/A	N/A	N/A	192	Live
G25	1	0	0	171	Live
G26	1	0	0	115	Live
G27	N/A	N/A	N/A	164	Death

Supplementary Table 2 The list of over expressed genes in each subgroup.

Subgroup A	Subgroup B	Subgroup C
RP11-284N8.3	PPBP	MIEN1
HHEX	ACTB	S100A11
MBTD1	FLNA	RPS23
MALAT1	TMSB4X	RPS16
PELI2	AP001189.4	RPS5
DENND4C	PF4	RPL18
NEAT1	CCL5	GSTP1
FAM65C	CLU	RPS8
HERC1	NRGN	S100A6
NFKBIA	UBXN11	RPL7A
CNST	TREML1	RPS19
RNF103	SH3BGRL3	RPS14
MOB1B	MYL9	RPLP0
ARRDC3	TLN1	RPL19
ZFP36	RP5-977B1.11	KRT19
DYRK1A	NCOA4	RPS24
RP11-38P22.2	GPX1	RPL23
EGF	FERMT3	RPL28
MGEA5	TAGLN2	RPS4X
GTF2B	CMTM5	RPL5
RP11-363E7.4	RABGAP1L	RPL8
IFNGR1	RSU1	NQO1
TMEM140	GGTA1P	RPS6
CLK1	NDUFAF3	RPL13A
STON2	AGBL5	HSP90AA1
FRMD3	RHEB	HSPA8
CXCL2	ARPC1B	RPLP1
PLEKHF2	ITGB5	RPS2
TXNIP	CRBN	GNB2L1
CD69	COTL1	ANXA1

Supplementary Table 3 Comparison of total duration of therapy before CTC collection of gastric cancer patients who received first-line therapy.

	Total duration of first-line therapy before CTC collection (day)	P value
Patient with subgroup A (n = 5)	168.8 ± 127.6	0.33
Patient without subgroup A (n = 4)	91.0 ± 97.3	

Supplementary Table 4 Primers used for qRT-PCR

Primer	Sequence (5'→)
GAPDH Fw	CCACCACACTGAATCTCCCC
GAPDH Rv	TGGTACATGACAAGGTGCGG
ACTB Fw	GAAGGCTTTTGGTCTCCCTG
ACTB Rv	TTCAACTGGTCTCAAGTCAGTGT
TP53 Fw	CTGCAAGCACATCTGCATTTTC
TP53 Rv	ACACAGGTGGCAGCAAAGTTTTA
GUSB Fw	AAGTGCCTCCTGGACTGTTC
GUSB Rv	CCACCTTTAGTGTTCCCTGCTA
CDKN2B Fw	TGAGTCCTGCTTCTAGCTCCA
CDKN2B Rv	GTACAAACCTTGGTAATGTCTTAGG
PIAS3 Fw	CTCATGGCCCTGTAGTTAGGC
PIAS3 Rv	AGATTGGGAAGGAGGGCACA
CYCS Fw	AGTGCAGGTTGTATGTGTCTA
CYCS Rv	ACTAGCCCAGTGGTCCTTTC
LAMA2 Fw	ACCCCAGGAAGAGTCTGTCAA
LAMA2 Rv	GGAGTCTGAATTAGCACCATCTG
AKT3 Fw	AAGGTCGTGGTGCGTTGTG
AKT3 Rv	ACCCAGCATGCCACAATCTG
COL4A2 Fw	TGACCGCCTTAATCTCGTGTC
COL4A2 Rv	ATTAAAAACAGCAGCGTGGGC
