# Supplementary material for: Transcriptomic profiling of single circulating tumor cells provides insight into human metastatic gastric cancer
Source: Commun Biol. 2022 Jan 11;5:20. doi: 10.1038/s42003-021-02937-x (PMC8752828; doi:10.1038/s42003-021-02937-x)
Supplement: Supplementary file 2 — Description of Additional Supplementary Files [file 42003_2021_2937_MOESM2_ESM.pdf]

## Description of Additional Supplementary Files

**File name:** Supplementary Data

**Description:**

Supplementary Data 1: Summary of patients.

Supplementary Data 2: The results of gene ontology analysis of genes strongly expressed in subgroup A.

Supplementary Data 3: The results of gene ontology analysis of genes strongly expressed in subgroup B.

Supplementary Data 4: The yield of single-cell whole transcriptome amplification (WTA) products. Single cells were subjected to Quartz-seq based WTA and WTA products were measured by BioAnalyzer High-sensitivity DNA kit (Agilent). The data corresponded to Figure 1B.

Supplementary Data 5: Raw Cq value of single-cell WTA products. The data corresponded to Fig. 1C.

Supplementary Data 6: Log-scaled RPM values of targeted genes in single cells. The data corresponded to Fig. 3A.

Supplementary Data 7: Gene expression score of epithelial and mesenchymal genes calculated by addModuleScore. The data corresponded to Fig. 3B.

Supplementary Data 8: Raw read count of single-cell RNA-seq data. The data corresponded to Fig. 4BC and Supplementary figure 6.

Supplementary Data 9: The number of detected gene from single CTCs in subgroup A and B. The data corresponded to Fig. 5A.

Supplementary Data 10: The yield and average length of single-cell whole transcriptome amplification (WTA) products. Single cells were subjected to Quartz-seq based WTA and WTA products were measured by BioAnalyzer High-sensitivity DNA kit (Agilent). The data corresponded to Supplementary Figure 1.

Supplementary Data 11: The yield of whole transcriptome amplification (WTA) products. Total RNA were subjected to Quartz-seq based WTA and WTA products were measured by BioAnalyzer High-sensitivity DNA kit (Agilent). The data corresponded to Supplementary Figure 2.

Supplementary Data 12: The yield and average length of single-cell whole transcriptome amplification (WTA) products. Single cells were isolated by GCM or micromanipulation. Then single cells were subjected to Quartz-seq based WTA and WTA products were measured by

BioAnalyzer High-sensitivity DNA kit (Agilent). The data corresponded to Supplementary Figure 3.

Supplementary Data 13: Percentage of mitochondrial gene in single-cell RNA-seq data. The data corresponded to Supplementary Figure 4.

Supplementary Data 14: The data showed nCount and nFeature value of single cells. The data corresponded to Supplementary Figure 5.
