# Supplementary material for: Transcriptomic profiling of single circulating tumor cells provides insight into human metastatic gastric cancer
Source: Commun Biol. 2022 Jan 11;5:20. doi: 10.1038/s42003-021-02937-x (PMC8752828; doi:10.1038/s42003-021-02937-x)
Supplement: Supplementary file 4 — Reporting Summary [file 42003_2021_2937_MOESM4_ESM.pdf]

nature research

Corresponding author(s): Tomoko Yoshino

Last updated by author(s): Oct 31, 2021

Reporting Summary

Nature Research wishes to improve the reproducibility of the work that we publish. This form provides structure for consistency and transparency in reporting. For further information on Nature Research policies, see our Editorial Policies and the Editorial Policy Checklist.

Statistics

For all statistical analyses, confirm that the following items are present in the figure legend, table legend, main text, or Methods section.

n/a	Confirmed	
☐	☒	The exact sample size (n) for each experimental group/condition, given as a discrete number and unit of measurement
☐	☒	A statement on whether measurements were taken from distinct samples or whether the same sample was measured repeatedly
☐	☒	The statistical test(s) used AND whether they are one- or two-sided<br>*Only common tests should be described solely by name; describe more complex techniques in the Methods section.*
☐	☒	A description of all covariates tested
☐	☒	A description of any assumptions or corrections, such as tests of normality and adjustment for multiple comparisons
☐	☒	A full description of the statistical parameters including central tendency (e.g. means) or other basic estimates (e.g. regression coefficient) AND variation (e.g. standard deviation) or associated estimates of uncertainty (e.g. confidence intervals)
☐	☒	For null hypothesis testing, the test statistic (e.g. F, t, r) with confidence intervals, effect sizes, degrees of freedom and P value noted<br>*Give P values as exact values whenever suitable.*
☒	☐	For Bayesian analysis, information on the choice of priors and Markov chain Monte Carlo settings
☒	☐	For hierarchical and complex designs, identification of the appropriate level for tests and full reporting of outcomes
☒	☐	Estimates of effect sizes (e.g. Cohen's d, Pearson's r), indicating how they were calculated

Our web collection on statistics for biologists contains articles on many of the points above.

Software and code

Policy information about availability of computer code

Data collection	LuminaVision (Mitani corp.) for microscopy imaging.
Data analysis	Seurat v2.0 and R 3.6 for single-cell transcriptome data analysis.

For manuscripts utilizing custom algorithms or software that are central to the research but not yet described in published literature, software must be made available to editors and reviewers. We strongly encourage code deposition in a community repository (e.g. GitHub). See the Nature Research guidelines for submitting code & software for further information.

Data

Policy information about availability of data

All manuscripts must include a data availability statement. This statement should provide the following information, where applicable:

- Accession codes, unique identifiers, or web links for publicly available datasets
- A list of figures that have associated raw data
- A description of any restrictions on data availability

The CTC sequencing data presented in this article are deposited in the DDBJ Sequence Read Archive (DRA011720). All data are available from the corresponding author upon reasonable request.

Field-specific reporting

Life sciences study design

All studies must disclose on these points even when the disclosure is negative.

Sample size	We used 27 patient samples for the validation of our proposed system.
Data exclusions	CTC with no expression of GAPDH genes were excluded from the transcriptome analysis.
Replication	We used a human lung cancer cell line (NCI-H1975) and gastric cancer cell lines (NCI-N87, AGS and SNU-1) for reproducibility test in Fig. 2, Fig. S1 and Fig. S3.
Randomization	N/A
Blinding	N/A

Reporting for specific materials, systems and methods

We require information from authors about some types of materials, experimental systems and methods used in many studies. Here, indicate whether each material, system or method listed is relevant to your study. If you are not sure if a list item applies to your research, read the appropriate section before selecting a response.

Materials & experimental systems

n/a	Involved in the study
☐	☒ Antibodies
☐	☒ Eukaryotic cell lines
☒	☐ Palaeontology and archaeology
☒	☐ Animals and other organisms
☐	☒ Human research participants
☒	☐ Clinical data
☒	☐ Dual use research of concern

Methods

n/a	Involved in the study
☒	☐ ChIP-seq
☒	☐ Flow cytometry
☒	☐ MRI-based neuroimaging

Antibodies

Antibodies used	Anti-CD45 antibody (Hitachi Chemical)
Validation	Antibody was verified by vendor.

Eukaryotic cell lines

Policy information about cell lines

Cell line source(s)	NCI-H1975, NCI-N87, AGS and SNU-1 were purchased from American Type Culture Collection.
Authentication	The cell lines were authenticated.
Mycoplasma contamination	Mycoplasma contamination was done regularly and proved to be negative.
Commonly misidentified lines (See ICLAC register)	N/A

Human research participants

Policy information about studies involving human research participants

Population characteristics	For validation of proposed system using human blood, study subjects were consisted of healthy donors. For CTC analysis, study subjects were consisted of women and men with metastatic gastric cancer at any stage of treatment. Clinical features of these patients are provided in Supplementary Data 1.
Recruitment	Healthy donors were recruited through the Tokyo University of Agriculture and Technology. Subjects with metastatic gastric cancer were recruited through the Tokyo Metropolitan Cancer and Infectious Diseases Center, Komagome Hospital. Informed consent was obtained from all healthy donors and patients.
Ethics oversight	For validation study, the study protocols were approved by the Institutional Review Board at Tokyo University of Agriculture and Technology (Approval code: No. 30-10). For CTC analysis, the study protocols were approved by the Institutional Review Board at Tokyo Metropolitan Cancer and Infectious Diseases Center Komagome Hospital (Approval Number: 1441).

Note that full information on the approval of the study protocol must also be provided in the manuscript.
